# Identifying falsified COVID-19 vaccines by analysing vaccine vial label and excipient profiles using MALDI-ToF mass spectrometry

**DOI:** 10.1038/s41541-024-01051-3

**Published:** 2025-01-30

**Authors:** Benediktus Yohan Arman, Rebecca Clarke, Tehmina Bharucha, Laura Gomez Fernandez, John Walsby-Tickle, Michael Deats, Sara Mosca, Qianqi Lin, Sneha Banerjee, Shrikrishna R. Chunekar, Kundan D. Patil, Sunil Gairola, Susanna Dunachie, Hamid A. Merchant, Robert Stokes, Rutendo Kuwana, Alexandrine Maes, Jean-Philippe Charrier, Fay Probert, Céline Caillet, Pavel Matousek, James McCullagh, Paul N. Newton, Nicole Zitzmann, Bevin Gangadharan

**Affiliations:** 1 Department of Biochemistry, University of Oxford, OX1 3QU Oxford, UK; 2 Kavli Institute for Nanoscience Discovery, University of Oxford, OX1 3QU Oxford, UK; 3 Department of Chemistry, University of Oxford, OX1 3TA Oxford, UK; 4 Medicine Quality Research Group, NDM Centre for Global Health Research, Nuffield Department of Medicine, University of Oxford, Oxford, OX3 7LG UK; 5 Mahidol-Oxford Tropical Medicine Research Unit, Faculty of Tropical Medicine, Mahidol University, Bangkok, 10400 Thailand; 6 Infectious Diseases Data Observatory, Centre of Tropical Medicine & Global Health, Nuffield Department of Medicine, University of Oxford, Oxford, OX3 7LG UK; 7 Central Laser Facility, Research Complex at Harwell, STFC Rutherford Appleton Laboratory, UKRI, Harwell Campus, Oxford, OX11 0QX UK; 8 Serum Institute of India Pvt. Ltd., 212/2, Hadapsar, Pune, 411028 India; 9 Department of Microbiology and Infectious Diseases, Oxford University Hospitals NHS Foundation Trust, Oxford, OX3 9DU UK; 10 NIHR Oxford Biomedical Research Centre, Oxford University Hospitals NHS Foundation Trust, Oxford, OX3 9DU UK; 11 Department of Bioscience, School of Health, Sport and Bioscience, University of East London, Water Lane, London, E15 4LZ UK; 12 Agilent Technologies LDA UK, Becquerel Avenue, Didcot, OX11 0RA UK; 13 Regulation and Safety Unit, Regulation and Prequalification Department, Access to Medicines and Health Products Division, World Health Organization (WHO), Geneva, Switzerland; 14 Department of Microbiology Research & Development, bioMérieux, Marcy l’Étoile, France; 15 Present Address: Hybrid Materials for Opto-Electronics Group, Department of Molecules and Materials, MESA+ Institute for Nanotechnology, Molecules Center and Center for Brain-Inspired Nano Systems, Faculty of Science and Technology, University of Twente, 7500AE Enschede, the Netherlands

**Keywords:** Public health, Drug regulation

## Abstract

The rapid development and worldwide distribution of COVID-19 vaccines is a remarkable achievement of biomedical research and logistical implementation. However, these developments are associated with the risk of a surge of substandard and falsified (SF) vaccines, as illustrated by the 184 incidents with SF and diverted COVID-19 vaccines which have been reported during the pandemic in 48 countries, with a paucity of methods for their detection in supply chains. In this context, matrix-assisted laser desorption ionisation-time of flight (MALDI-ToF) mass spectrometry (MS) is globally available for fast and accurate analysis of bacteria in patient samples, offering a potentially accessible solution to identify SF vaccines. We analysed the COVISHIELD™ COVID-19 vaccine; falsified versions of which were found in India, Myanmar and Uganda. We demonstrate for the first time that analysis of spectra from the vaccine vial label and its adhesive could be used as a novel approach to detect falsified vaccines. Vials tested by this approach could be retained in the supply chain since it is non-invasive. We also assessed whether MALDI-ToF MS could be used to distinguish the COVISHIELD™ vaccine from surrogates of falsified vaccines and the effect of temperature on vaccine stability. Both polysorbate 80 and L-histidine excipients of the genuine vaccine could be detected by the presence of a unique combination of MALDI-ToF MS peaks which allowed us to distinguish between the genuine vaccines and falsified vaccine surrogates. Furthermore, even if a falsified product contained polysorbate 80 at the same concentration as used in the genuine vaccine, the characteristic spectral profile of polysorbate 80 used in genuine products is a reliable internal marker for vaccine authenticity. Our findings demonstrate that MALDI-ToF MS analysis of extracts from vial labels and the vaccine excipients themselves can be used independently to detect falsified vaccines. This approach has the potential to be integrated into the national regulatory standards and WHO’s Prevent, Detect, and Respond strategy as a novel effective tool for detecting falsified vaccines.

## Introduction

The rapid development of vaccines against coronavirus disease 2019 (COVID-19) was a remarkable success in biomedical research marked by the supply and distribution worldwide, with billions of doses administered^1^. However, the vital importance but inequitable global distribution of COVID-19 vaccines has been associated with increasing concerns about the occurrence and impact of substandard and falsified (SF) vaccines^2–4^. According to the World Health Organization (WHO), approximately 10% of medical products distributed in low- and middle-income countries (LMIC) are either substandard or falsified^5,6^. This concerning estimate suggests a significant but neglected risk, potentially leading to higher rates of illness and death, as well as eroding public trust in healthcare systems and economic harm. In light of the growing recognition of the importance of vaccines in the fight against diseases, this issue becomes even more significant.

Substandard or ‘out of specification’ products are authorised medical products that fail to meet either their quality standards or specifications, or both. If the vaccine cold chain is not maintained in the supply chain (e.g. if left at ambient temperature which can be above 45 °C in some countries) this is likely to result in a poor-quality substandard vaccine. In contrast, falsified products refer to products that deliberately or fraudulently misrepresent their identity, composition or source^6^.

SF medical products pose a significant threat to health as they are of poor quality, unsafe and/or ineffective^7^. Moreover, there is inadequate recognition of the scale of the problem or of the need to establish appropriate risk analysis, monitoring and intervention systems. In the public domain, there have been reports of 184 incidents of diverted and SF COVID-19 vaccines from 48 countries and most cases of vaccine falsification have been in LMIC^8^. In one of these 184 incidents alone, 2,500 people were wrongly injected with saline as a fake instead of the genuine COVID-19 vaccine^9^. In other cases, 6,000 vials of falsified COVISHIELD™ COVID-19 vaccine (equivalent to 60,000 doses) were seized in Varanasi^10^ and a member of the Indian parliament (along with a few hundred other individuals) was injected with falsified COVISHIELD™^11^. Other COVID-19 vaccines have also been falsified including the Oxford AstraZeneca vaccine (in Iran)^12^ and Pfizer-BioNTech Comirnaty® vaccine (in Poland, Mexico and Iran)^13,14^. Before the pandemic, multiple non-COVID-19 vaccines had also been falsified including vaccines for cholera (Dukoral® in Bangladesh), rabies (Verorab® in Philippines) and meningitis (Mencevax® in Niger)^15^.

There are no readily available devices currently being used in the supply chain to screen for SF vaccines. Such devices are required to empower inspectors and enforcement agencies conducting risk-based post-market surveillance, that could be integrated into the national regulatory standards and WHO’s Prevent, Detect, and Respond strategy^2^. The risk of SF vaccines in the pandemic spawned increased interest, with the evaluation of near-infrared^16^, spatially-offset Raman spectroscopy (SORS)^17^ and lateral flow test devices^18^.

The Vaccine Identity Evaluation Collaboration (https://www.cghr.ox.ac.uk/research/medicine-quality-research-group/mqrg-projects/vaccine-identity-evaluation) has been evaluating a diverse range of screening devices^17,18^ and here we describe the application of matrix-assisted laser desorption ionisation–time-of-flight mass spectrometry (MALDI-ToF MS) which provides spectral information where peaks correspond to the molecular weights of ionised molecules (e.g. proteins and other compounds). MALDI-ToF MS is widely available throughout the world including in some LMIC where they are used in clinical microbiology laboratories for the identification and speciation of micro-organisms^19^ and other specific purposes, such as to identify proteins in influenza virus vaccines^20^. We have recently established a MALDI-ToF MS method to differentiate genuine non-Covid vaccines from falsified vaccine surrogates^21^. However, there has been no published literature reporting the use of MALDI-ToF MS to analyse vaccine vial labels, the spectral peak distribution of polysorbate in vaccines or the other constituents of COVID vaccines to help with their authentication.

In this study, we report the application of MALDI-ToF MS to distinguish genuine COVISHIELD™ (a COVID-19 vaccine manufactured by Serum Institute India Pvt. Ltd.) from a range of falsified vaccine surrogates. COVISHIELD™ is a recombinant non-replicating chimpanzee adenovirus viral vector (ChAdOx1) vaccine encoding the severe acute respiratory syndrome coronavirus 2 (SARS-CoV-2) Spike glycoprotein^22^. Falsified COVISHIELD™ vaccine has been identified in Africa (Uganda) and South-East Asia (India and Myanmar)^23^. In these cases, the criminals changed the labels in the following ways: falsifying the expiry date (Uganda), stating an incorrect volume of 2 mL with 4 doses instead of 5 mL with 10 doses (India), and falsifying the batch number with an incorrect spelling of vaccine brand as COVISHELD (Myanmar). It is not in the public domain what the criminals used for the falsified labels in the three countries. However, from the photos in the WHO Medical Product Alert^23^, it appears as though the labels could have been self-adhesive office labels with the label information printed with an office colour printer.

Since WHO have reported changes in the label for falsified COVISHIELD™ vials which have been seized, we evaluated the use of MALDI-ToF MS to analyse extracts from the COVISHIELD™ labels to see if we could acquire unique spectral fingerprint for the genuine label and its adhesive. The analysis of labels helps to differentiate batches but most importantly is non-invasive and therefore allows the tested vaccine vials, found to be authentic, to be retained in the supply chain. MALDI-ToF MS was also used to analyse the vaccine liquid using our recently reported method^21^ and we have for the first time applied MALDI-ToF MS for a COVID-19 vaccine and evaluated if the method can detect heat exposure degradation (i.e. substandard) vaccines. Furthermore, we show that the distribution of polysorbate 80 spectral peaks acts as an internal marker for vaccine authenticity due to their characteristic peak profiles and significant spectral variations between polysorbate 80 manufacturing sources. Two different MALDI-ToF MS systems were used in this study to compare results - a bioMérieux VITEK® MS and a Bruker Biotyper® Sirius. Both devices are commonly used in hospital microbiology laboratories worldwide making them potentially readily available for vaccine screening programs.

## Results

### Inter- and intra-batch analyses of COVID-19 vaccine

**Fig. 1:**
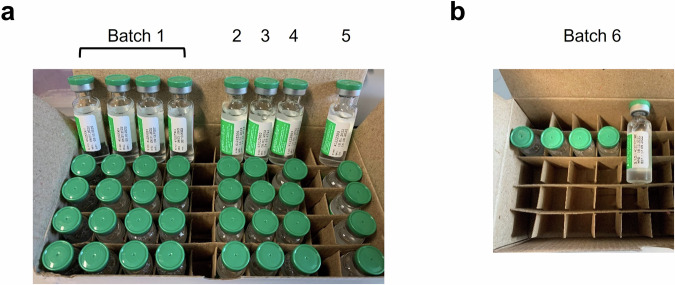
The genuine Serum Institute India (SII) COVISHIELD™ COVID-19 vaccine samples used in the study. Batches of vaccine samples from (**a**) Hadapsar factory and (**b**) Manjari factory were analysed.

**Fig. 2:**
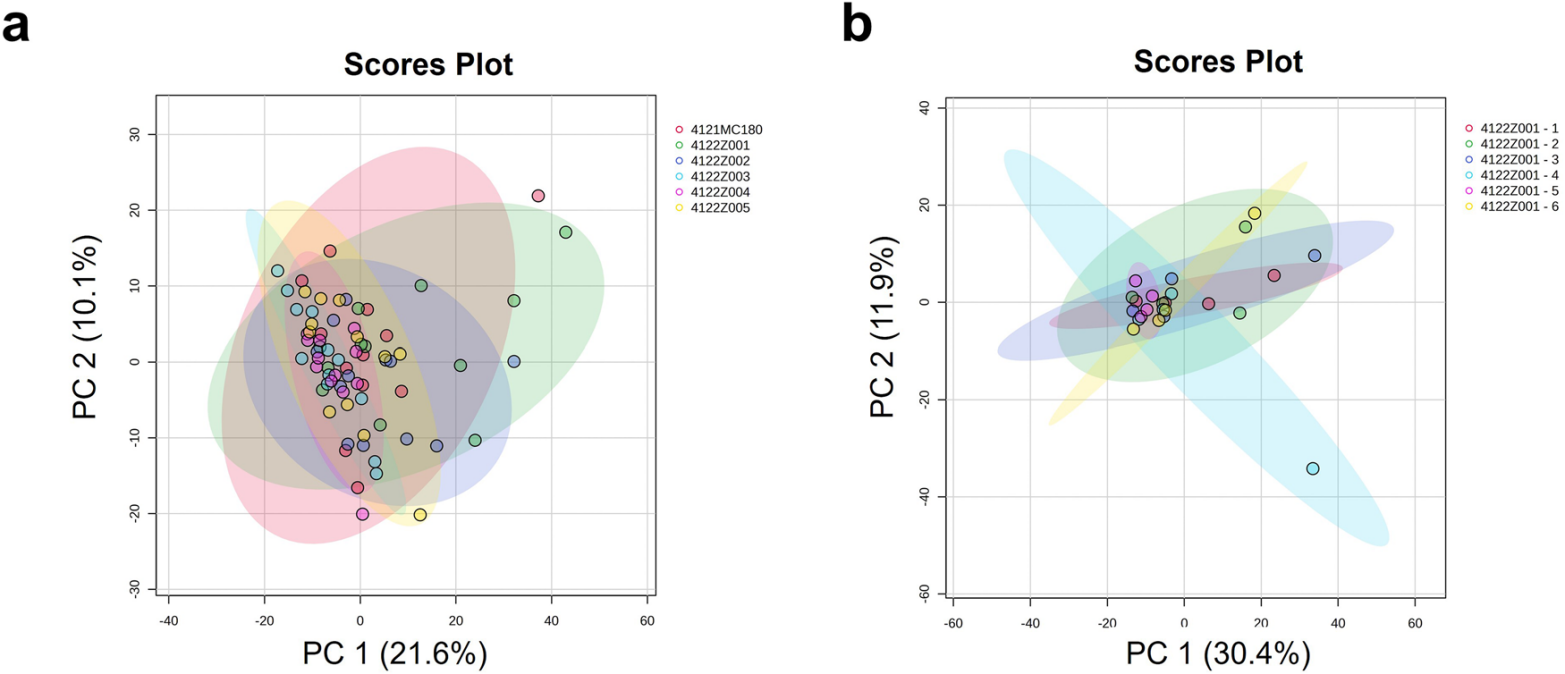
Multivariate analyses of the MALDI-ToF MS spectra generated from COVISHIELD™ COVID-19 vaccine samples. PCA score plots were generated for **a** Six different batch numbers of COVISHIELD™ vaccine (inter-batch analysis, *n* = 3) for each batch number, with four technical replicates for each n, generating a total of 12 spectra replicates; and **b** Six vaccine vials of the same batch number 4122Z001 (intra-batch analysis, *n* = 3). Each vial had 4 technical replicates. Spectra generated using Biotyper MALDI-ToF MS at 0-900 m/z.

The inter- and intra-batch analyses were performed on genuine COVISHIELD™ vaccine vials (Fig. 1 and Table 1). Using the peak list generated by Biotyper, principal component analysis (PCA) score plots of the first two most significant principal components showed no differences between batches (Fig. 2a) and between vials with the same batch number (Fig. 2b). These can be observed by the overlapping 95% confidence regions, shown as coloured elliptic areas. Similar results were generated from the analysis of spectra from the Vitek-MS (Supplementary Fig. 1).

**Table 1:** COVISHIELD™ vaccine samples batch numbers from Hadapsar factory (Batch 1-5) and Manjari factory (Batch 6) in India, used in the study

Batch group	Vaccine batch number	Number of vials
Batch 1	4122Z001	20
Batch 2	4122Z002	5
Batch 3	4122Z003	5
Batch 4	4122Z004	5
Batch 5	4122Z005	5
Batch 6	4121MC180	5

### Detection of vaccine excipient masses using MALDI-ToF MS

**Fig. 3:**
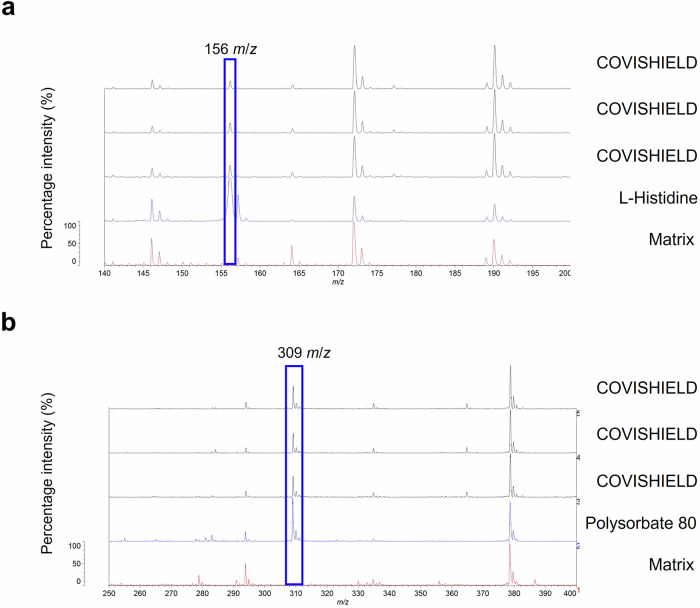
Identification of COVISHIELD™ COVID-19 vaccine constituents at 0-900 *m/z* using the Vitek-MS. **a** L-histidine was identified at 156 *m/z.* **b** A low mass peak of polysorbate 80 was detected at 309 *m/z*. The COVISHIELD™ spectra from three different vials of vaccine batch 4122Z001 are shown and compared to L-histidine, polysorbate 80 and matrix spectra.

**Fig. 4:**
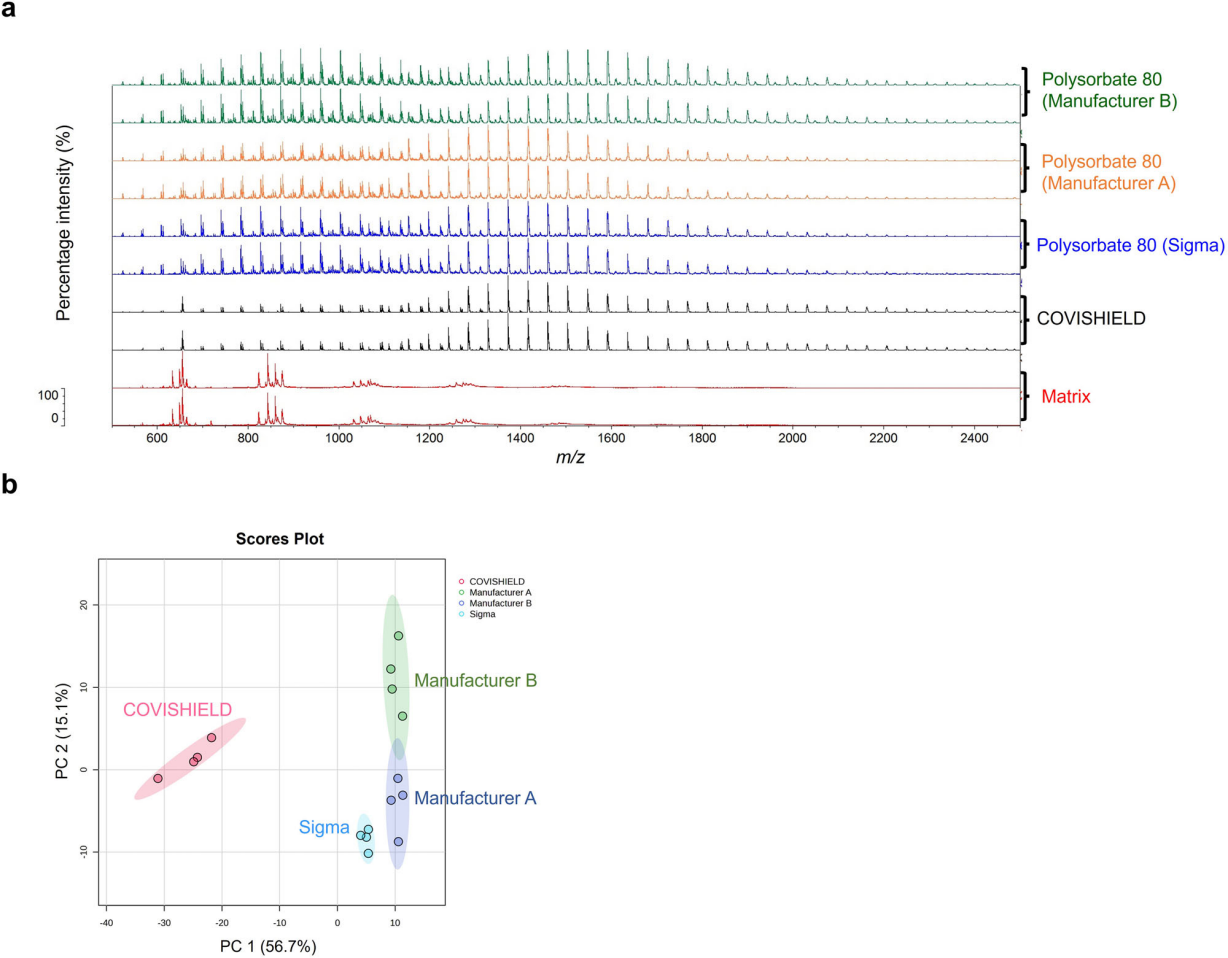
Vitek-MS spectra comparison between COVISHIELD™ and polysorbate 80 from three different manufacturers. **a** Spectra over the 500-2,500 *m/z* mass range showing different profiles of polysorbate 80 with the most dominant evenly spaced peaks between 600 and 2200 *m/z*. **b** PCA scores plot showing the distinct grouping of COVISHIELD™ and polysorbate 80 from three different manufacturers. Samples were run with four technical replicates for each and only two representative spectra are shown.

In our attempt to identify COVISHIELD™ vaccine constituents using MALDI-ToF MS, visual inspection of the spectra at 0-900 *m/z* revealed the presence of a peak at 156 *m/z* which corresponded to the nominal mass for the excipient L-histidine (Fig. 3a) that was not detected in the matrix spectra. L-histidine was made up in water as a standard and analysed by MALDI-ToF MS. As expected, a peak at 156 *m/z* was observed which aligned with the 156 *m/z* for the COVISHIELD™ vaccine. The spectra for the excipient polysorbate 80, which was made up in water as a standard, showed a unique peak at 309 *m/z* (Fig. 3b) and as a series of evenly spaced peaks in the mid-mass range 700-2500 *m/z* (Fig. 4a and Supplementary Fig. 2a) and in the high mass range 2000-20,000 *m/z* (Supplementary Fig. 2b) that were not observed in the matrix spectra but were seen in the genuine COVISHIELD™ vaccine. In the high mass range, most of these evenly spaced peaks for polysorbate 80 in COVISHEILD™ were seen from 2000 to 4,000 *m/z* (Supplementary Fig. 3).

### Differentiation of genuine COVISHIELD™ COVID-19 vaccine and surrogates of falsified vaccines

**Fig. 5:**
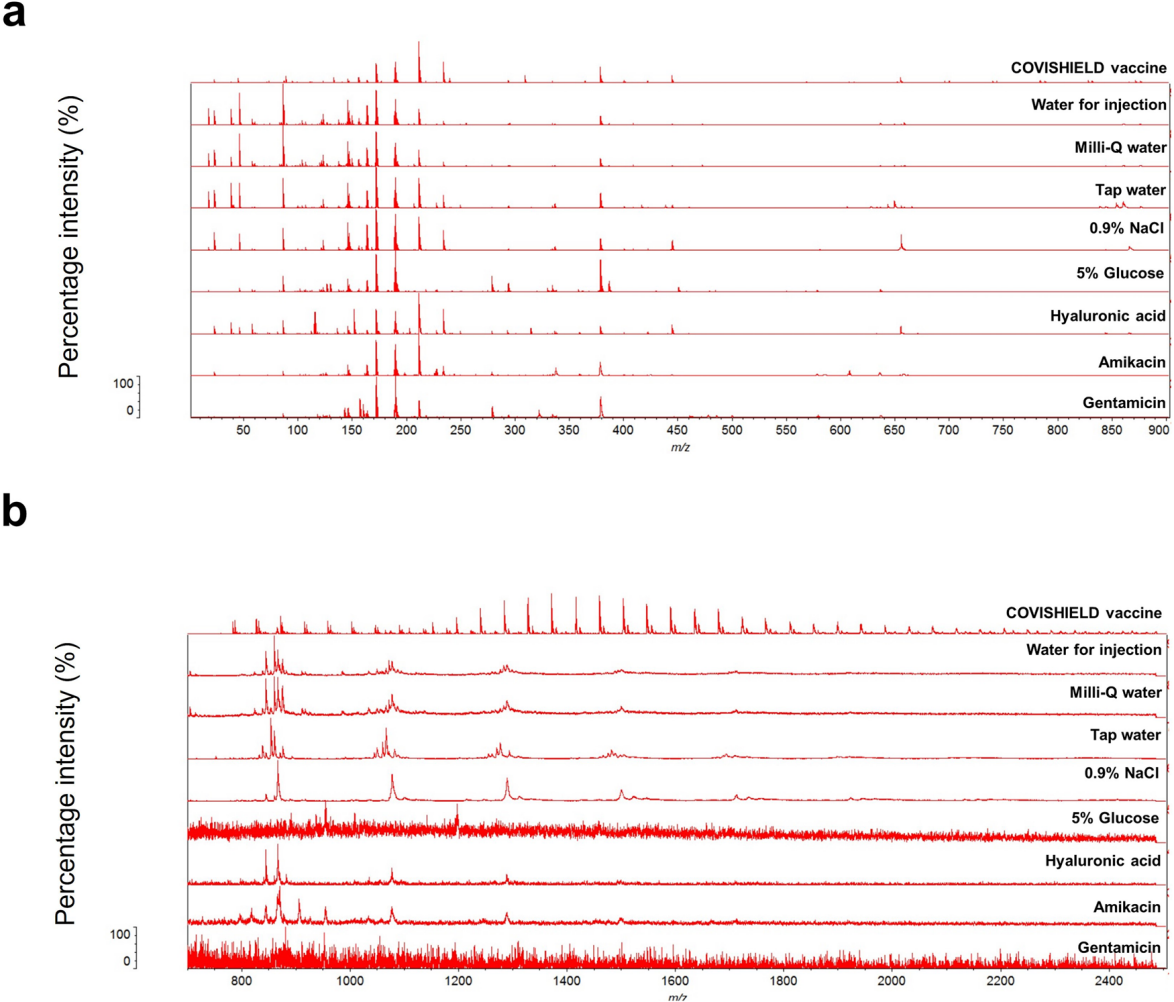
Vitek-MS spectra for the COVISHIELD™ vaccine and eight falsified vaccine surrogates. Spectra were generated at (**a**) 0–900 m/z and (**b**) 700–2500 m/z mass ranges.

**Fig. 6:**
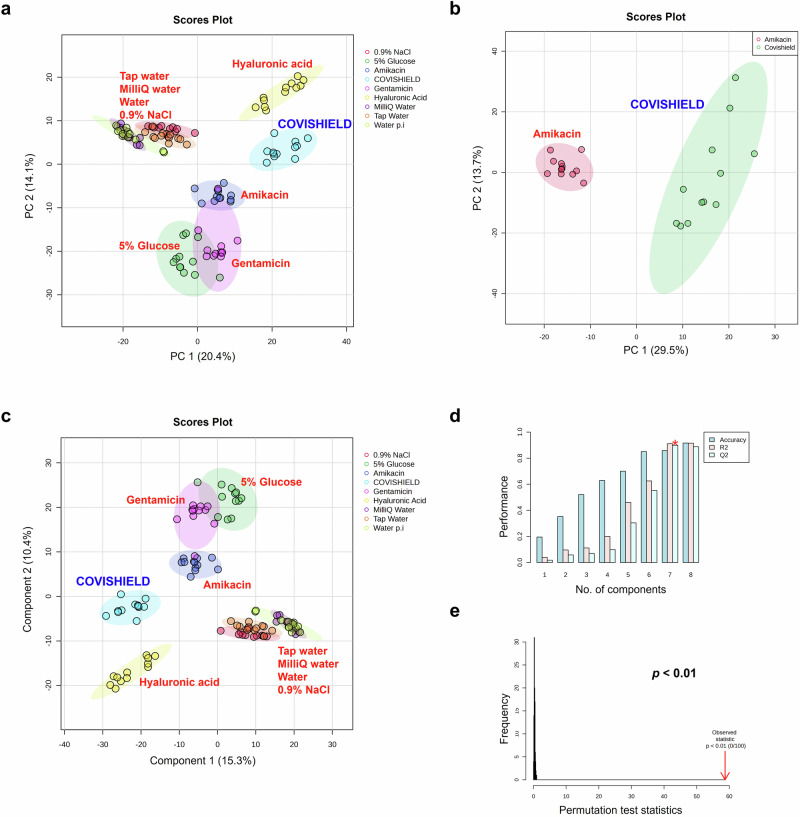
Multivariate statistical analyses of the MALDI-ToF MS spectra generated from genuine COVISHIELD™ COVID-19 vaccine samples and eight common falsified vaccine surrogates. PCA score plots were generated from analysis of Vitek-MS peak list data over the 0-900 m/z mass range (*n* = 3 with four technical replicates for each n) for (**a**) COVISHIELD™ vaccine as compared to eight common falsified vaccine constituents and (**b**) COVISHIELD™ compared to Amikacin only, along with (**c**) PLS-DA score plot, **d** PLS-DA cross-validation results, and **e** PLS-DA permutation analysis. The elliptical area reflects the region within 95% confidence interval of the measurement.

MALDI-ToF MS was used to differentiate between genuine COVISHIELD™ vaccine and surrogates of falsified vaccines (Table 2). Different spectral peaks could be visually observed in the low 0–900 (Fig. 5a) and 700–2500 *m/z* (Fig. 5b) mass ranges when comparing the spectra of the genuine vaccine alongside eight surrogates. Although some peaks overlapped in the spectra between the genuine COVISHIELD™ vaccine and the surrogates at 0-900 *m/z*, there were also peaks unique to COVISHIELD™, such as the peaks mentioned earlier at 156 and 309 *m/z*, specific for L-histidine and polysorbate 80, respectively (Fig. 3a and b). COVISHIELD™ could be readily distinguished from the other surrogates at the mid-mass range (700-2500 *m/z*) (Fig. 5b) and also at the 2000-20,000 *m/*z range that is routinely used in microbial identification (Supplementary Fig. 3). The spectral profile of polysorbate 80 was reproducible among the six batches of COVISHIELD™ vaccine (Supplementary Fig. 4). However, the peaks for polysorbate 80 from other manufacturers were different and could be well separated by PCA (Fig. 4b). The PCA score plots of the first two principal components showed a distinct separation of the COVISHIELD™ vaccine from all falsified vaccine surrogates (Fig. 6a), without any overlap in the 95% confidence regions. This was supported by partial least squares discriminant analysis (PLS-DA) with cross-validation and permutation testing (Fig. 6c-e) which demonstrated perfect separation of all groups (Supplementary Table 1). To confirm the robustness of the PLS-DA model and investigate its potential to identify vaccine surrogates from previously unseen data, an external validation was applied by splitting the data into training (80%) and test (20%) sets. This model was able to distinguish between COVISHIELD™ vaccine from all falsified vaccine surrogates with 100% accuracy in both the training and the external validation test sets with a 90/10 split (Supplementary Table 1). In addition to this 90/10 split, a 70/30 split was also carried out also showing perfect accuracy for the genuine vaccine (data not shown).

**Table 2:** The falsified vaccine surrogates used for MALDI-ToF MS analysis

Vaccine surrogates	Manufacturer	Batch/part number	Composition	Remarks
0.9% w/v sodium chloride injection	Demo S.A Pharmaceutical Industry	24598/0002	0.9% w/v NaCl in water for injection	Surrogate for falsified COVID-19 vaccines intercepted in China and India (Mumbai)^8^
5.0% w/v D-glucose	B/Braun	03551/0059	D-glucose 5.0% w/v Glucose solution prepared in distilled water	Surrogate for falsified COVID-19 vaccines intercepted in the Philippines^8^
Amikacin, 250 mg/ml	Hospira (used for the Sirius Biotyper)	05015997122159	250 mg/ml amikacin sulphate, sodium citrate, sodium metabisulphite and water for injection	Surrogate for falsified COVID-19 vaccines intercepted in India^8,34^
	MA Holder Tillomed Laboratories Ltd. (used for the bioMérieux Vitek-MS)	11311/0604	250 mg/ml amikacin sulphate, sodium metabisulfite, sodium citrate dihydrate, sulfuric acid and water for injection	
Gentamicin, 40 mg/ml	Demo S.A.	05208063001339	40 mg/ml gentamicin sulphate, 1.60 mg/ml sodium metabisulfite, disodium edetate	Surrogate for falsified non-COVID vaccines intercepted in Indonesia^8^
Hyaluronic acid	Guangzhou Ailian Cosmetic Co Ltd.	QB/T 2660	Anti-wrinkle serum containing water, glycerine, propylene glycol, methylisothiazolinone, bis(hydroxmethyl) imidazolidinyl urea, iodopropynyl butylcarbamate, disodium EDTA, xanthan gum, sodium hyaluronate	Surrogate for falsified COVID-19 vaccines intercepted in Poland^8,41^ (the precise formulation and form of intercepted hyaluronic product unknown apart from it being reported being an anti-wrinkle formulation)
Tap water	Biochemistry/Chemistry Research Laboratory, Oxford	N/A	Tap water	Tap water from the building water facility
Milli-Q water	Merck Millipore	N/A	Water from a Milli-Q Direct 8 water purification system	Purified double-distilled water
Water for injection	Demo S.A Pharmaceutical Industry	24598/001	Sterile water for preparation of a medicine intended for injection or infusion	Water for injection in plastic ampoules

**Fig. 7:**
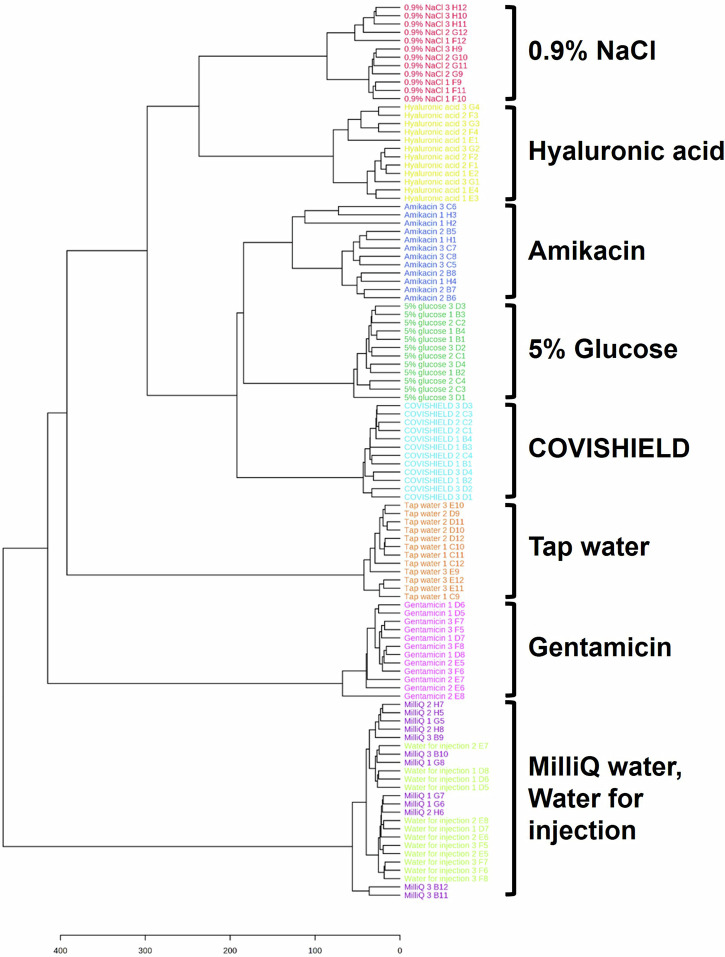
Dendrogram analysis of the MALDI-ToF MS spectra (*n* = 3, with four technical replicates for each n) for the COVISHIELD™ vaccine as compared to eight surrogates of falsified vaccine constituents. This classification is based on data from the Vitek-MS instrument at 0-900 *m/z*.

Amikacin has been reported to be used as falsified COVISHIELD™ COVID-19 vaccines intercepted in India during the pandemic^8^. When analyzing only COVISHIELD™ and amikacin, the PCA scores plots could differentiate between them (Fig. 6b and Supplementary Fig. 5b). This was also supported by the PLS-DA scores plot with good cross-validation (high accuracy, R2, and Q2 scores) and significant permutation test (*p* < 0.05) results (Fig. 6c–e) and perfect accuracy of external validation confusion matrices (Supplementary Table 1). Dendrogram analysis revealed the clustering of COVISHIELD™ samples into a monophyletic group (Fig. 7 and Supplementary Fig. 6). Other surrogates also clustered into separate groups in the dendrogram, except for the Milli-Q water and water for injection since both are purified water and, as expected, had similar spectra (Fig. 6a, Fig. 6c, Fig. 7 and Supplementary Fig. 5a).

### Effect of temperature degradation on genuine COVID-19 vaccine

**Fig. 8:**
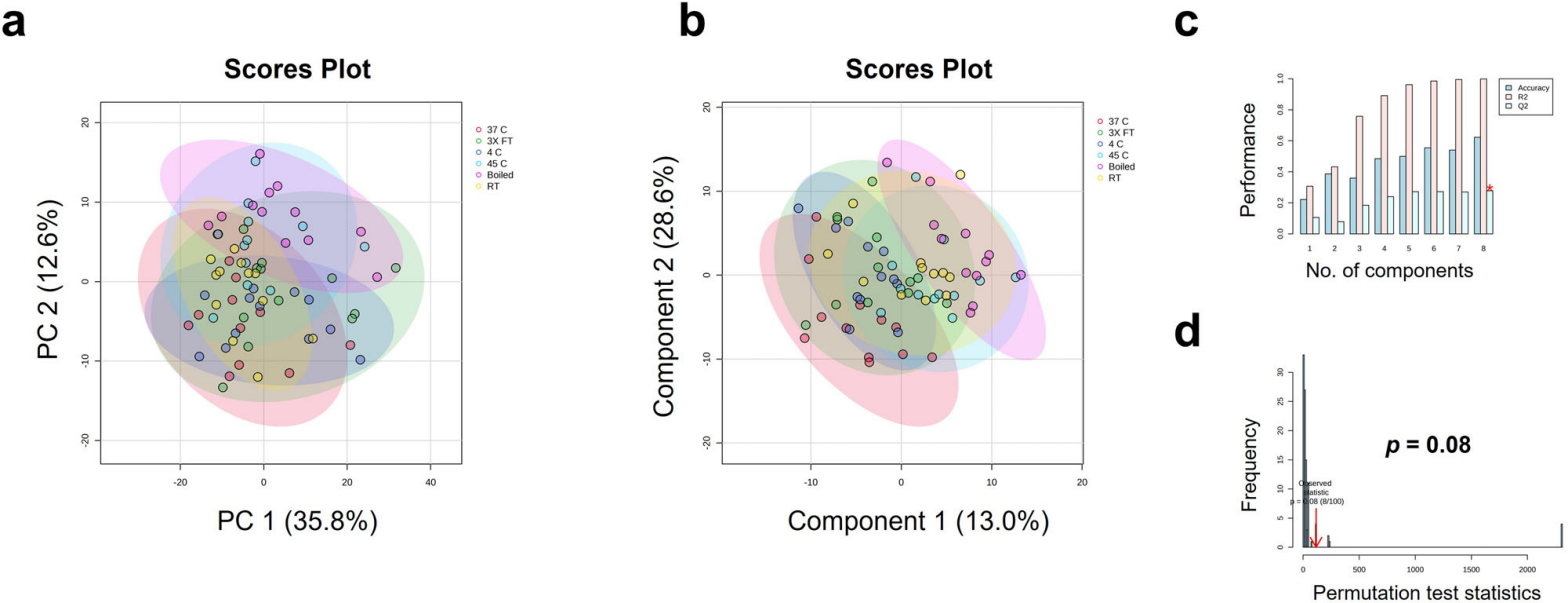
Vitek-MS analysis at 0-900 *m/z* comparing COVISHIELD™ vaccine vials exposed to freeze thaw cycles and different temperature conditions (three vials run (*n* = 3), with four technical replicates for each n). **a** PCA score plot, **b** PLS-DA score plot, **c** PLS-DA cross-validation results, and **d** PLS-DA permutation analysis.

In order to explore vaccine degradation, vaccine samples were exposed to different temperature conditions followed by comparison using MALDI-ToF analysis. Multivariate analysis of vaccine spectra showed no difference between degradation conditions, as depicted as the plotting of samples with overlapping 95% confidence region of PCA (Fig. 8a). Further, PLS-DA resulted in a non-significant model (Fig. 8b) with low cross-validation scores (Fig. 8c) and non-significant permutation test (Fig. 8d). The high R2 of this model and accuracy of the model on training data (Supplementary Table 2) is consistent with overfitting as confirmed by external validation which resulting in an accuracy of only 75% on the test set (Supplementary Table 2). No significant differences were observed when analysing spectra from the mid-mass (700-2500 *m/z*) and high-mass (2000-20,000 *m/z*) ranges (Supplementary Fig. 7).

### Analysis of vaccine vial labels

**Fig. 9:**
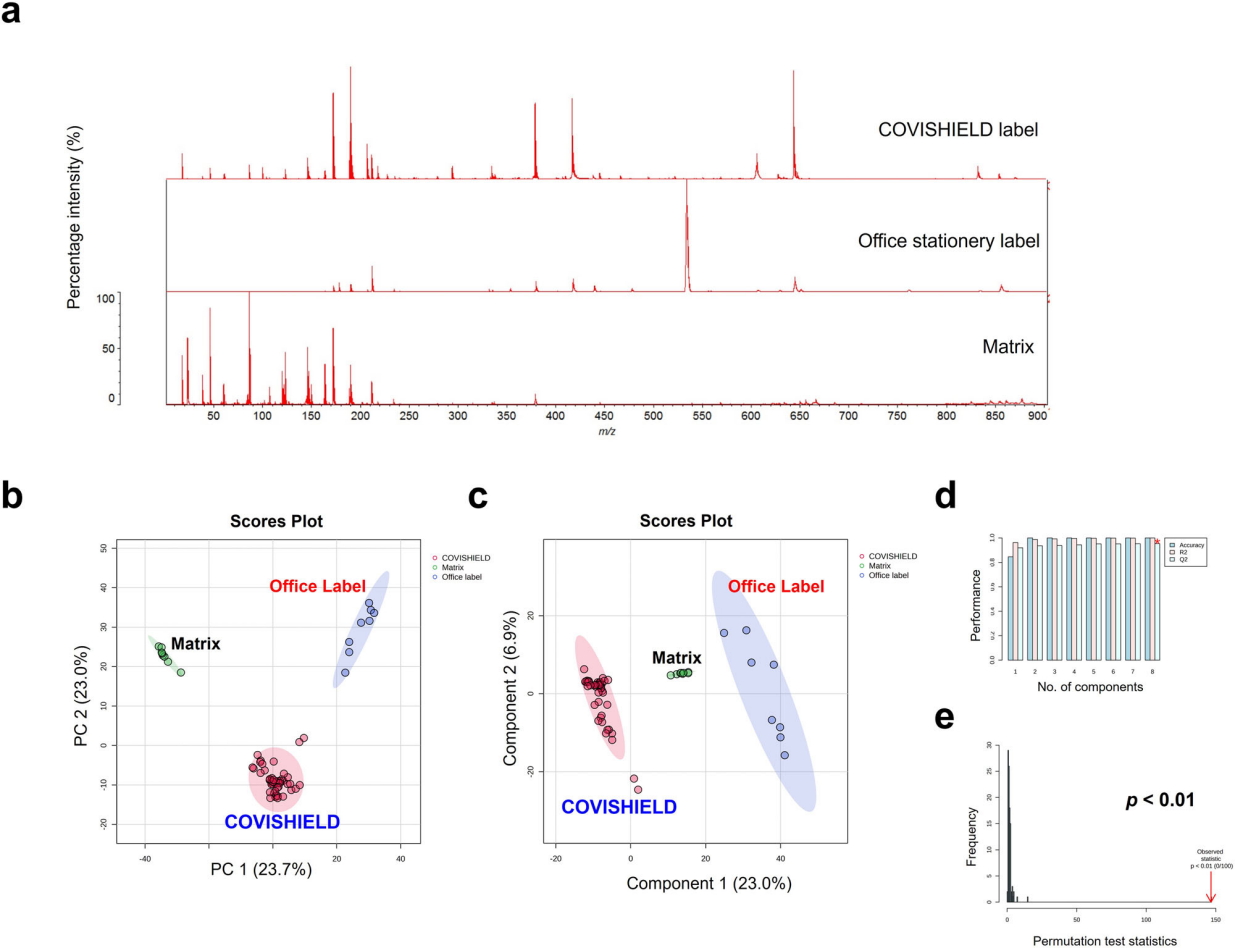
Vitek-MS analysis of labels over the 0-900 *m/z* mass range comparing COVISHIELD™ label extracts from six batches (*n* = 6), an office stationery label (*n* = 2), and the CHCA matrix as a background control (*n* = 1), with four technical replicates for each n. **a** The 0–900 *m/z* representative spectra of label extracts and matrix, **b** multi-variate analysis PCA score plot, **c** PLS-DA score plot, **d** PLS-DA cross-validation results, and **e** PLS-DA permutation analysis.

**Fig. 10:**
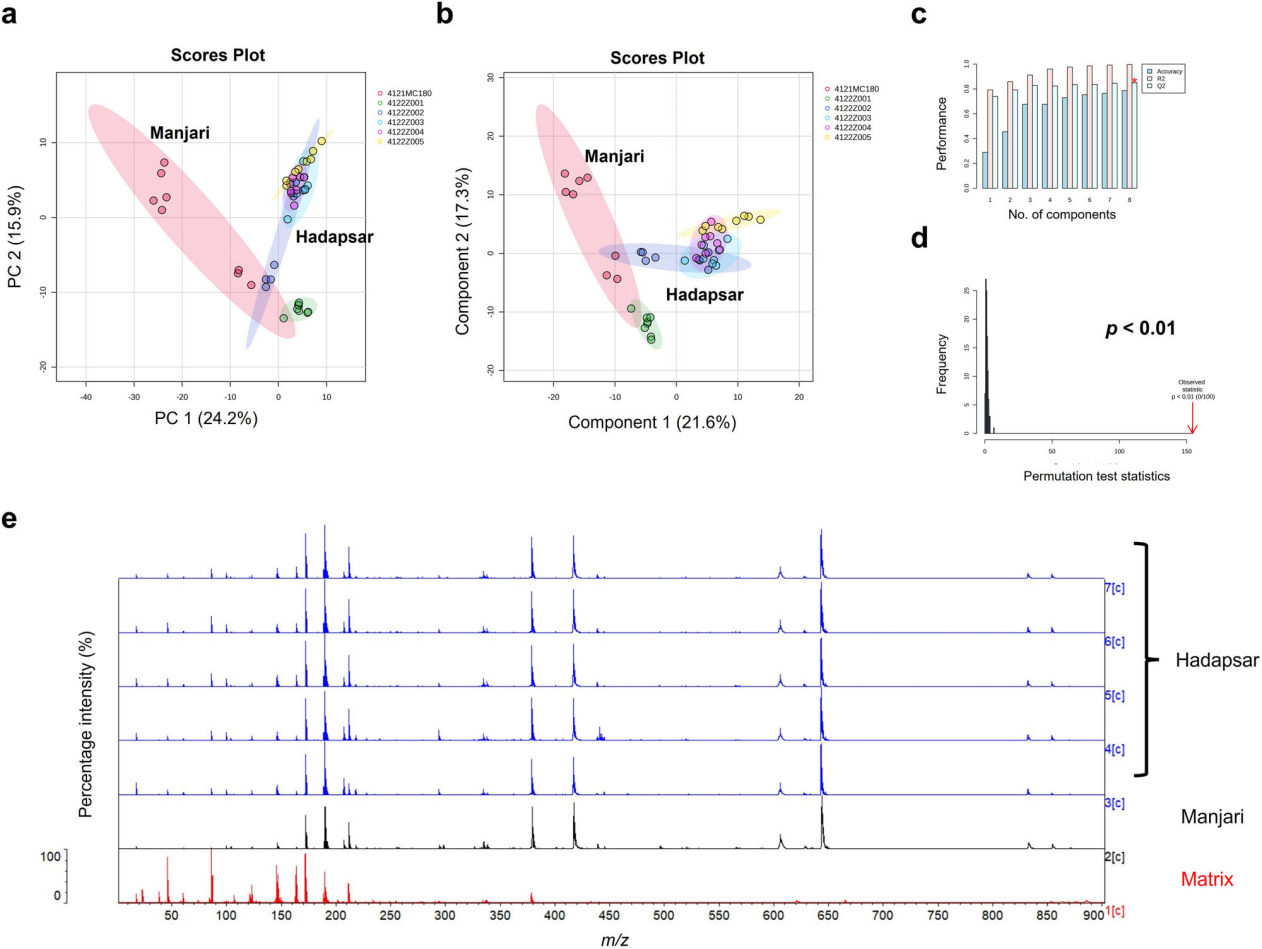
Vitek-MS analysis over the 0-900 *m/z* mass range of COVISHIELD™ vial label extracts from six different batches (*n* = 8 technical replicates per batch) manufactured in Hadapsar and Manjari factories (Table 1). **a** PCA score plot, **b** PLS-DA score plot, **c** PLS-DA cross-validation results, **d** PLS-DA permutation analysis, and **e** the 0–900 *m/z* representative spectra of label extracts from Hadapsar (five different batches) and Manjari (one batch), compared to matrix.

We investigated proof-of-concept that MALDI-ToF MS analysis could also be used to identify differences in chemical composition between vaccine vial label extracts from genuine COVISHIELD™ labels and an office stationery label (Fig. 9). The spectra for genuine COVISHIELD™ labels was found to be remarkably different to the office stationery label and differences could easily be identified simply by visualising the spectra (Fig. 9a) and without needing to perform any statistical analysis of the spectral data. In addition, PCA (Fig. 9b) and PLS-DA (Fig. 9c) scores plots of the genuine and stationery label spectra showed clear separation with no overlap of the 95% confidence regions and significant cross-validation and permutation results (Fig. 9d, e). This was confirmed by external validation which demonstrated that the PLS-DA model was able to distinguish between vaccine labels with 100% accuracy in the training and test sets using a 90/10 split (Supplementary Table 3). In addition to this 90/10 split, a 70/30 split was also carried out also showing perfect accuracy for the genuine labels (data not shown). When comparing between peaks of genuine COVISHIELD™ label and office stationery label, t-test statistical analysis revealed a total number of 395 significant (*p* < 0.05) peaks with the peak at 534 *m/z* as the variable of importance in projection with the highest score based on PLS-DA analysis, shown as the base peak for office stationery label (Fig. 9a). PCA could further distinguish between labels used in different batches of COVISHIELD™ vials produced in Hadapsar and Manjari factories, although some overlapping of the 95% confidence regions were observed (Fig. 10a). In addition, this was supported by a robust PLS-DA model, shown by high accuracy, R2, and Q2 scores and a significant permutation test (Fig. 10b–d) with perfect accuracy of the model on the training and test data sets using a 90/10 split (Supplementary Table 4). In addition to this 90/10 split, a 70/30 split was also carried out also showing perfect accuracy for the different manufacturing sites (data not shown).

## Discussion

The lack of established quality assurance systems within the complicated meshwork of global medicinal product supply chains puts the world at risk of surges in SF medicines and vaccines^2^. This highlights the critical role of medicine regulatory authorities to prevent, detect and remove SF medicinal products at different points in the supply chain^24^ and such monitoring should be an intrinsic part of disease surveillance programs to safeguard public health^25^.

MALDI-ToF MS instruments measure the mass-to-charge ratio (*m/z*) of individual analyte ions and can be used for the analysis of complex mixtures with minimal sample preparation and rapid turnaround (data for each sample is acquired in only a few seconds)^26^. Crucially, thousands of these MS instruments are widely available^27^ in hospital microbiology laboratories globally, for bacterial identification, and therefore provide a potential accessible analytical network for local vaccine analysis. Although the MALDI-ToF instruments used in hospitals are of low resolution in comparison to other mass spectrometers, they were capable of differentiating genuine vaccines from all falsified vaccine surrogates tested and genuine labels from an office stationery label. A limitation of some MALDI-ToF instruments in hospitals is that only the 2000–20,000 *m/z* range is routinely used in microbial identification. Although this is a limited range, it does cover the mass range for the polysorbate 80 observed in this study (Supplementary Fig. 2b). Peaks such as histidine at 156 *m/z* and polysorbate 80 at 309 *m/z* (Fig. 3) would not be observed if only the routinely used mass range of 2000–20,000 *m/z* is used. However, many MALDI-ToF mass spectrometers in hospitals are already capable of analysing a wide mass range (0–500 *m/z*) covering these peaks and all other instruments set to analyse the 2000–20,000 *m/z* can be upgraded to analyse the wide mass range.

We demonstrate how MALDI-ToF MS with machine learning analysis of the results can be used to verify genuine vaccine vial labels and detect key excipients in the COVISHIELD™ vaccine and use them as indicators of the genuine vaccine. In addition, we confirmed that the PLS-DA model was robust, suggesting it could be used to predict new, previously uninvestigated vaccine samples, irrespective of origin, and confirm if it is a genuine or falsified vaccine as shown by the high accuracy of the external validation results in both training and test sets (Supplementary Table 1).

Falsified COVISHIELD™ vaccine and vial labels have been identified in Uganda, India and Myanmar^23^. Since labels on the COVISHIELD™ vials have been falsified, we compared the extracts of a self-adhesive office label with the genuine COVISHIELD™ labels. MALDI-ToF MS analysis of label extracts with their adhesives showed that COVISHIELD™ vaccine labels had distinct spectral profiles (Fig. 9a). PCA scores plot showed distinct clustered groups for the genuine labels, office stationery label and matrix background (Fig. 9b). In addition, a significant (permutation test *p*-value < 0.05) PLS-DA model was generated (Fig. 9c) that was able to distinguish between label types with 100% accuracy in the test set (Supplementary Table 3). A limitation is that it was not possible to obtain the actual falsified COVISHIELD™ vaccine label. From the photos published on the WHO alert^23^, it appears as though the criminals used an office label on the falsified COVISHIELD™ vaccine vials and printed the text on the label using an office printer. Therefore, an office stationery label was similar to what had been used on the falsified vaccine vials. While the label tested was not an actual falsified label, the data shows that MALDI-ToF is capable of non-invasively detecting falsified vaccines. The spectra of only one office label were analysed for proof-of-concept. Although only one office label was compared to several genuine COVISHIELD™ labels, the permutation test confirms that the separation observed is unlikely to have resulted by chance.

Different score plots were observed between the two manufacturing sites (Fig. 10), inferring the possibility of either slightly different label materials/adhesives between factories or that the same materials/adhesives were used but MALDI-ToF MS is sensitive enough to detect a different batch of label material or adhesive. Since MALDI-ToF was able to differentiate genuine labels from the Hadapsar and Manjari factories (possibly due to a different batch of adhesive/label), it would be able to differentiate a genuine label and any non-genuine label used by criminals. We have also analysed the label extracts of other COVID-19 vaccines (data not shown due to non-disclosure agreements in place) which showed unique spectra, different to the genuine COVISHIELD™ label, indicating that vaccine labels from different vaccine manufacturers have their own mass spectral fingerprints which could be used to non-invasively check authenticity. The criminal reuse of genuine vials filled with non-vaccine liquids would not be detected. However, these results illustrate a promising additional non-invasive method to detect falsification of vaccines and the approach could be applied to analyse the labels or packaging of all medicines as well as other items known to be falsified (e.g. perfume bottles). In this study, MALDI-ToF MS was found to be capable of detecting even differences in the batches of adhesive used between the Manjari and Hadapsar sites (Fig. 10). Although the spectra were different, the dominant peaks were the same suggesting that the difference is due to a different batch of adhesive/label (Fig. 10e). These dominant peaks could be included in a reference spectral library to test for authenticity. In addition, there are less intense peaks which vary between batches and can help to confirm if a spectrum matches with that of a reference label from the same batch. While it is required for vaccine manufacturers to disclose the list of excipients in vaccines, details for the label are proprietary information making it almost impossible for criminals to reproduce a label with identical MALDI-ToF MS spectra without knowing the label material, label manufacturer and the exact composition (formulation) of the adhesive. Furthermore, they would need to know the precise concentration of ingredients used in the specific adhesive batch they are attempting to copy since the technique was sensitive enough to differentiate between adhesive batches (Fig. 10). Many vaccine manufacturers also have security features on vaccine labels which are difficult to replicate but the unique MALDI-ToF spectra of the label extracts (molecular fingerprints) add a considerably higher level of security that cannot be replicated by falsifiers. If a vaccine manufacturer changes the label or its adhesive, a label from a genuine batch could be used as a reference to compare to a test label. The advantage of analysing the label is that it is non-invasive and the vials can be retained in the supply chain. However, where a vaccine fails the label check and remains suspect, the vaccine liquid should be additionally analysed.

MALDI-ToF MS can also be utilised to establish a molecular profile for a vaccine^20,21^. Vaccine constituents such as polysorbate 80 (with evenly spaced peaks mainly between 500-2500 *m/z*) and L-histidine (at 156 *m/z* corresponding to [histidine + H]^+^) are known to be seen by positive ionisation mass spectrometry^28,29^ and their presence was confirmed in the COVISHIELD™ vaccine using authentic standards. Peaks corresponding to L-histidine (Fig. 3a) and polysorbate 80 (Figs. 3b and 4) are therefore possible markers of the COVISHIELD™ vaccine. We expect that the 309 *m/z* peak, unique to the authentic COVISHIELD™ vaccine, could be the C18:1 ester of polysorbate as previously reported^30^.

Polysorbate 80 consists of a population of ethoxylated structures with different masses observed in MALDI-ToF MS spectra as evenly distributed peaks resulting from random polymerisation. The peaks are spaced apart in the spectra by 44 Da, equal to the mass of ethylene oxide (C_2_H_4_O; 44.05 g/mol). These evenly spaced peaks were observed predominantly between 500-2500 *m/z* (Fig. 4a and Supplementary Fig. 7). We analysed polysorbate 80 from multiple sources; a chemical supplier (Sigma) and two different online stores. The PCA analysis on spectra profiles was able to distinguish between polysorbate 80 from each manufacturer (Fig. 4b), suggesting that the distribution of polysorbate 80 peaks in the COVISHIELD™ vaccine could act as an internal marker for authenticity screening. While this study has focused on COVISHIELD™, a very large number of medicines contain polysorbates and therefore may be a good internal marker for authenticity. For example, COVID-19 (Paxlovid; nirmatrelvir-ritonavir)^31^, anti-malarial (Riamet; artemether-lumefantrine)^32^, and cholesterol-lowering (Lipitor; atorvastatin)^33^ drugs all contain polysorbate 80 and all have been associated with falsified products, the latter of which saw the largest recall of falsified medicines in the US^33^. The method could also be applied to drugs containing other polysorbates, such as polysorbate 20 and potentially for analysing other polymers commonly used as excipients in vaccines such as PEGylated lipids (e.g. polyethylene glycol 2000 dimyristoyl glycerol in Spikevax® COVID-19 vaccine, Moderna) or Triton X surfactants (e.g. octoxinol-9 in Quadrivalent Influenza vaccine, Sanofi).

As expected, MALDI-ToF MS was not able to detect the most dominant excipient sucrose since it is not usually seen with positive ionisation mass spectrometry. Also, as expected, ethanol in the vaccine was not visible since it evaporated off the MALDI target. Both sucrose and ethanol are excipients that we had previously detected using SORS by scanning unopened COVISHIELD^17^. However, SORS did not detect polysorbate 80 or L-histidine, highlighting the complementarity of the two techniques where SORS could be used as a screening technique and MALDI-ToF as a confirmatory analysis where an anomaly is identified.

MALDI-ToF MS of vaccines from vials of the same batch (intra-batch), different batches (inter-batch) and different manufacturing sites showed similar spectra with the same relative intensities among the excipient peaks for histidine and polysorbate 80. Therefore, falsified vaccines containing the same excipients but at different relative concentrations could be identified since the relative intensities among the histidine and polysorbate 80 peaks in falsified vaccines would differ from the relative intensities consistently observed with the genuine vaccine. If concentrations of histidine and polysorbate 80 were identical then differentiation may not be possible using only relative peak intensity ratios. In this case, the fingerprint of polysorbate 80, which we show is unique for a chemical manufacturer, would also need to be analysed. Furthermore, the PCA analysis yielded no significant differences in both intra- and inter-batch analyses (Fig. 2), reflecting the standard manufacturing quality implemented in both factories. MALDI-ToF and PCA analysis could distinguish COVISHIELD™ vaccine from the falsified vaccine surrogates (Fig. 6a), which strongly suggests that they could be used to detect falsified vaccines in risk-based post-market surveillance. In addition, the dendrogram classification grouped COVISHIELD™ samples into a monophyletic group separated from other surrogates (Fig. 7).

Substandard quality of a medicinal product may be generated from errors, negligence, or poor practice in manufacturing, procurement, regulation, transportation, or storage^7^. One of the potential risks in vaccine supply chains is the possibility of temperature excursions during transportation, or failure in maintaining the cold storage (cold chain) conditions, especially in countries where the ambient temperature is high. We therefore investigated the effect of freeze-thaw cycles and heat exposure on the spectra of the vaccine. Although the test set performed well (Supplementary Table 2), the high accuracy observed is likely due to chance and multivariate analysis was not able to detect any significant changes in heat-exposed samples (Fig. 8).

MALDI-ToF was unable to detect or check the stability of the active constituent of the vaccine and thus other methods (e.g. viral vector virus titration) are needed for this purpose. Although MALDI-ToF MS could not identify the active ingredients in the vaccine, it was still able to discriminate between simulated falsified vaccines and the genuine comparator vaccine. To detect active ingredients, we have recently proposed and evaluated repurposing rapid diagnostic tests (RDTs)^18^. This highlights the complementarity of both MALDI-ToF and RDTs to confirm if a vaccine is genuine or falsified. MALDI-ToF has the advantage of its distribution in most reference hospitals and many microbiology laboratories in the world, including in some LMIC, and therefore the technique is deployable in the field. Since thousands of MALDI-ToF mass spectrometers are in hospitals around the world, the vaccines could potentially be tested prior to vaccine administration in the same hospital.

Limitations of the study include that we did not have access to collected falsified vaccines. According to online news websites, the falsified COVISHIELD™ vaccine was found in a vaccination camp in Kolkata, India, where amikacin was used^34^. Using our approach, amikacin could be readily differentiated from the genuine COVISHIELD™ vaccine (Fig. 6b). Dilution of genuine vaccines is far less likely to be carried out by criminals since they would need to obtain the genuine vaccine to do this. We are not aware of any case where vaccines have been falsified by dilution and therefore have not tested this with COVISHIELD™.

As part of a future project, custom spectral libraries for genuine vaccines will be generated which could be distributed to international medicine regulatory agencies to use on MALDI instruments in their respective countries. Since no batch-to-batch variation was detected for the COVISHIELD™ vaccine (Fig. 2), a fixed spectral library could be used. Since differences were observed in the label spectra between manufacturing sites, a reference spectra would need to be generated from the label of a known genuine batch and then compared to a suspect label. Close liaison would be needed between vaccine manufacturers and regulatory authorities to ensure timely notification of changes in vaccine and label constituents.

In conclusion, we have demonstrated that MALDI-ToF MS spectral analysis combined with machine learning and multivariate analysis could be used for authenticating a COVID-19 vaccine. Excellent inter-batch reproducibility was observed for the COVISHIELD™ vaccine among six different batches and across two different manufacturing sites. Two excipients (L-histidine and polysorbate 80) in the COVISHIELD™ vaccine could be successfully detected and the spectra were easily differentiated, both visually and using multivariate analysis, from falsified vaccine surrogates. In addition, while analysis of the vaccine is destructive, we showed that the analysis of vaccine vial labels has the potential for use as a non-invasive method in vaccine authentication and could be applied to other medicine labels. Since label analysis is non-invasive, it would allow the tested vaccine vials to be retained in the supply chain post-screening.

## Methods

### Vaccine samples

A total of 45 vials of genuine Serum Institute India (SII) COVISHIELD™ COVID-19 vaccine, hereafter mentioned as COVISHIELD™ vaccine, were received for the study. The vials were acquired from five production batches at the Hadapsar factory (Batch 1-5) and the Manjari factory (Batch 6), both located in Pune, Maharashtra, India (Fig. 1 and Table 1). Three vials per batch were used for batch-to-batch analysis by MALDI-ToF MS with four technical replicates (a total of 12 replicates per batch).

The COVISHIELD™ vaccine is reported to contain the excipients L-histidine, L-histidine hydrochloride monohydrate, magnesium chloride hexahydrate, polysorbate 80, ethanol, sucrose, sodium chloride, and disodium edetate dihydrate (EDTA)^35^.

Vaccine samples were shipped in a temperature-controlled container and stored at 2-8°C upon arrival, according to the manufacturer’s recommendation, and kept on ice prior to spotting on the MALDI targets. All samples were analysed within their date of expiry.

### Single solutions of ionisable excipients

For the comparative vaccine excipient measurements, the following solutions were used: L-histidine in water (<15 mM; Sigma-Aldrich) and polysorbate 80 in water (<1% w/v Tween 80; Sigma-Aldrich). Commercially-available polysorbate 80 from two different online markets (www.thesoapery.co.uk, referred to as manufacturer A and www.cosmeticsmadeeasy.com, referred to as manufacturer B) were also prepared in the same <1% w/v concentration. Although not necessarily identical, these were in the same concentrations as those used in the authentic vaccines.

### Surrogate of falsified vaccines

We did not have access to falsified vaccine samples seized in supply chains. Hence, to assess the capability of the techniques to detect falsified COVID-19 vaccine products, surrogates for potential and actually intercepted falsified products were used (Table 2). Falsified vaccine constituents were identified from reports available in the public domain, both in the scientific and lay literature^8^.

### MALDI-ToF MS processing of samples

Sample processing was performed in three biological replicates (*n* = 3) with four technical replicates for each n. Samples were spotted onto disposable MALDI target plates (Vitek-MS P/N 410893 and Biotyper® Sirius P/N 1840375) compatible with VITEK® MS (bioMérieux), ‘Vitek-MS’, and Biotyper Sirius (Bruker) MALDI-ToF MS, ‘Biotyper’, instruments. Each technical replicate spot was produced by mixing an equal volume of sample and alpha-cyano-4-hydroxy-cinnamic acid (CHCA) matrix (CHCA/HCCA, Vitek-MS CHCA; P/N 411071, Bruker MS-CHCA solvent P/N 900666 and matrix P/N 8255344) then pipetting 2 µL of the mixture onto each replicate spot of the target plate using an ASSIST PLUS pipetting robot (INTEGRA Biosciences Ltd, Berkshire, UK). The sample spots were allowed to air dry at room temperature. All samples used were within their expiry dates at the time of experimental work. The mass ranges analyzed were 0-900, 700-2500 and 2000-20,000 *m/z* using our previously described method^21^.

### Degraded samples

To study the effect of temperature on the stability of the COVISHIELD™ vaccine, the following temperature conditions were investigated: vaccine vials stored at 4 °C (within the 2-8 °C manufacturer’s recommended storage condition) and vials stored at room temperature (recorded as 20 ± 1 °C) and at elevated temperature conditions of 37 °C, 45 °C and 100 °C (boiled). In addition, vials were exposed to three freeze-thawed cycles.

For each of these storage conditions, three unopened vials of COVISHIELD™ vaccine (*n* = 3, all from batch no. 4122Z001) were used. The 4 °C vials were refrigerated for 7 days, and the other vials were stored on bench at room temperature or in incubator ovens set at 37 or 45 °C for 7 days. The vials undergoing freeze-thaw cycles were frozen at –80 °C for 24 h and then thawed at room temperature (20 °C) for 1 h. The freeze-thaw cycle was repeated twice more. The boiled vials were placed inside a beaker of boiling water on a 100 °C hot plate for 10 min. All samples were stored at 4 °C after undergoing the above temperature conditions.

### Vaccine vial label analysis

A small piece of the vaccine vial label (approximately 2 × 2 mm) was cut, in duplicate, from different batches of the COVISHIELD™ vaccine vials (*n* = 3, with four technical replicates for each n). The self-adhesive office stationery labels (*n* = 2, with four technical replicates for each n) were used for comparison. Each piece of the label was then thoroughly mixed by vortexing, to help dissolve the adhesive into solution, with 10 μL of CHCA matrix, and then incubated for 10 min at room temperature. A volume of 2 μL of the extract was spotted in quadruplicate onto a MALDI plate and run to generate MS spectra at low mass (0–900 mass per charge, *m/z*), mid mass (700–2500 *m/z*) and high mass (2000–20,000 *m/z*) ranges^21^.

### Data analysis

Raw spectra from both instruments were visually analyzed and plotted using Shimadzu LaunchPad (for Vitek-MS data) and Bruker FlexAnalysis (for Biotyper data) software. Vitek-MS raw data files were acquired in mzXML format using Shimadzu LaunchPad software. Bruker CompassXport was used to acquire Biotyper data. Spectra (four spectra from four technical replicates for each N) were analysed using a workflow that combined MALDI-ToF with open-source machine learning and statistical analysis^21^. Briefly, data exploration was performed using R statistical software v.4.1.3 using packages MALDIQuant^36^, MALDIrppa, caret, lattice, factoextra, and dendextend while data visualisation was performed in MetaboAnalyst^37^. The resulting peak list from MALDIQuant was then analyzed using the ‘Statistical Analysis (one factor)’ module of MetaboAnalyst online software. Experimental data were uploaded as peak intensities in “Samples in columns (unpaired)” format. Data filtering was performed using an interquartile range (IQR) statistical filter to identify and remove variables that are unlikely to be of use when modelling the data. The data were normalised by sum and scaled using pareto scaling (mean-centered and divided by the square root of the standard deviation of each variable). Unsupervised principal component analysis (PCA) and supervised multivariate analysis with machine learning algorithm partial least squares-discriminant analysis (PLS-DA) were used to model the data. The PLS-DA cross-validation (CV) was performed using 8 maximum components to search and 5-fold CV method, and permutation testing validation using the separation distance method and 100 permutation numbers^21^. Hierarchical dendrogram clustering was performed using Euclidean distance measure and Ward clustering algorithm methods.

### PLS-DA external validation and confusion matrices

For each significant PLS-DA model generated, external validation was performed by randomly selecting 10% of the data to use as a pseudo external test set. Confusion matrices were produced as previously described^38^. In brief, PLS-DA models were generated using either (1) the entire data set or (2) 90% of each class randomly selected as a training set which was then used to predict both the training and test sets. The accuracy of the PLS-DA predictions relative to the true classification of each sample is then summarised in confusion matrices using R code generated in-house^39^ and the ropls^40^ package.

## Supplementary information

Supplementary information

## Data Availability

All data generated or analysed during this study are included in this published article and its supplementary information files.
