# Supplementary material for: Identifying falsified COVID-19 vaccines by analysing vaccine vial label and excipient profiles using MALDI-ToF mass spectrometry
Source: NPJ Vaccines. 2025 Jan 30;10:19. doi: 10.1038/s41541-024-01051-3 (PMC11782545; doi:10.1038/s41541-024-01051-3)
Supplement: Supplementary file 1 — Supplementary information [file 41541_2024_1051_MOESM1_ESM.pdf]

Supplementary files for Arman BY, *et al*. (2024) Identifying falsified COVID-19 vaccines by analysing vaccine vial label and excipients profiles using MALDI-ToF mass spectrometry

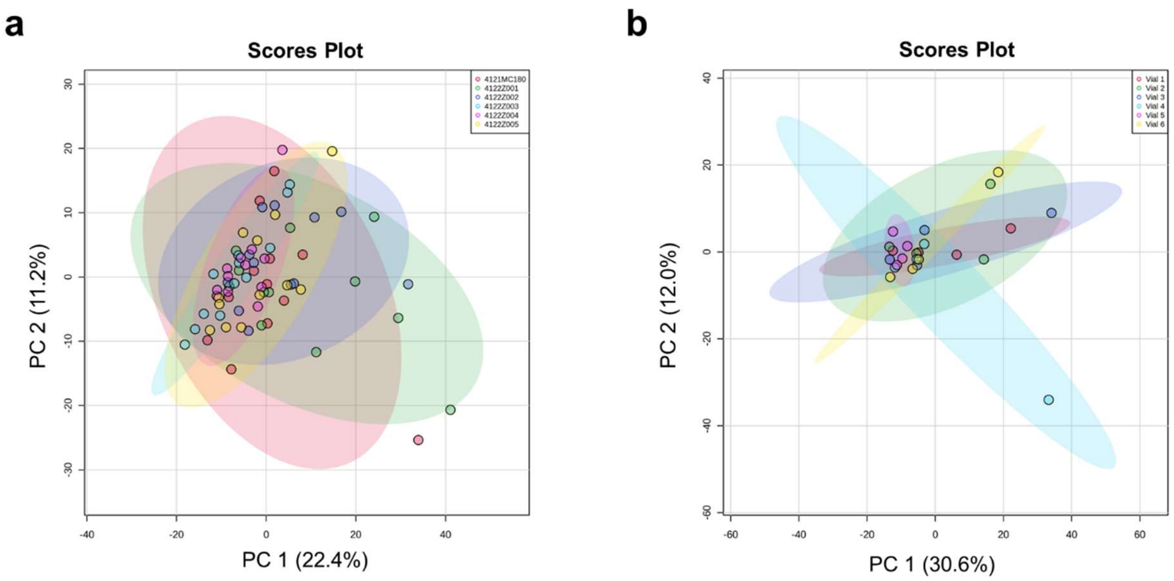

Supplementary Figure 1. PCA scores plot of the Vitek-MS spectra at 0-900 *m/z* for **a** Six different batch numbers of COVISHIELD™ vaccine (inter-batch analysis), each batch number contains 12 analytical replicates; and **b** Six vaccine vials of the same batch number 4122Z001 (intra-batch analysis), each vial has 4 analytical replicates.

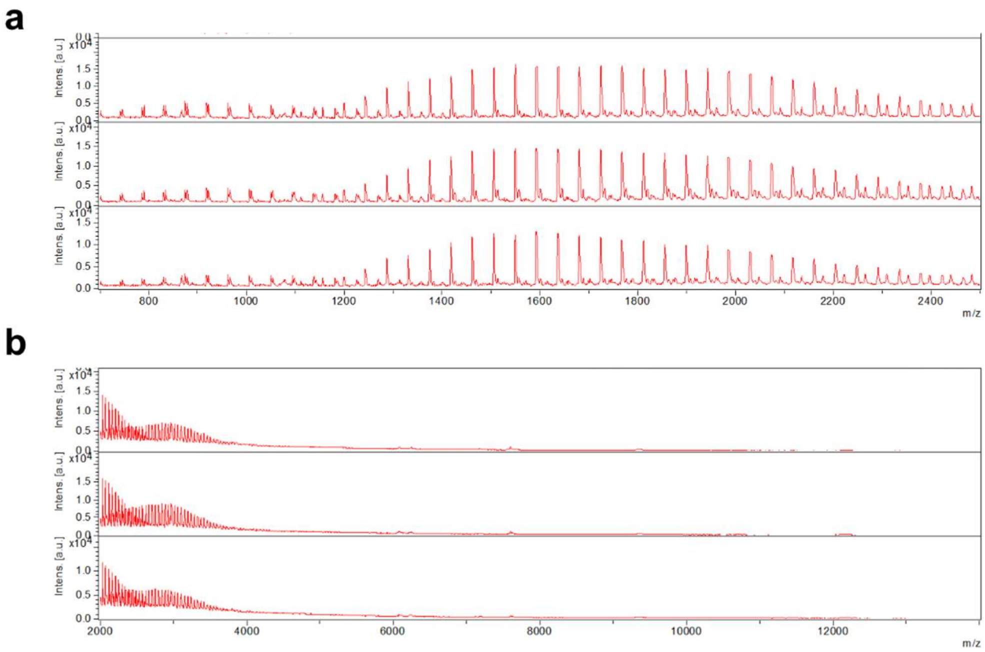

Supplementary Figure 2. Identification of polysorbate 80 in COVISHIELD™ vaccine as evenly spaced peaks. **a** Over the 700-2,500 *m/z* mass range and **b** Over the 2,000-20,000 *m/z* mass range (evenly spaced polysorbate 80 peaks between 2,000-4,000 *m/z*) using a Biotyper MALDI-ToF MS.

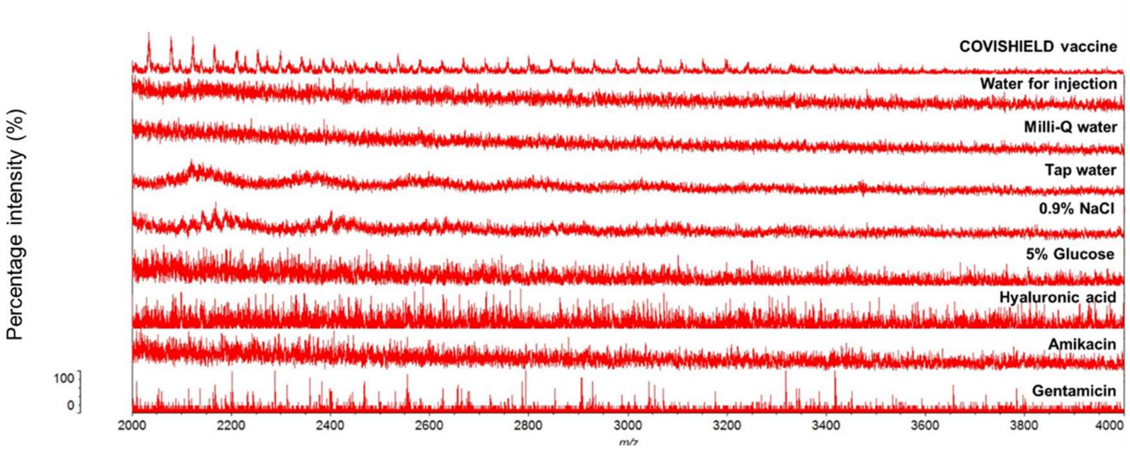

Supplementary Figure 3. Vitek-MS spectra for COVISHIELD™ vaccine and 8 falsified vaccine surrogates at 2,000-4,000 *m/z*.

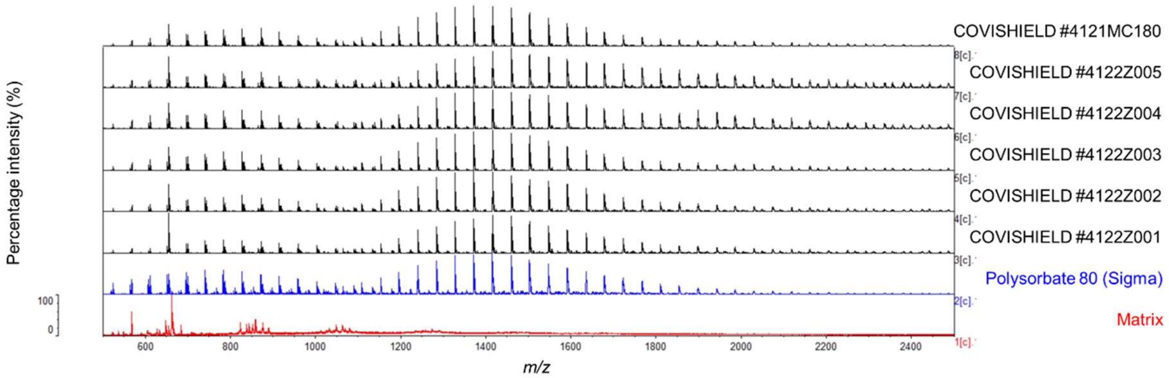

Supplementary Figure 4. Vitek-MS spectra (500-2500 *m/z)* comparing six batches of COVISHIELD™ vaccine and commercially-available polysorbate 80 (Sigma-Aldrich). Each sample was run with four technical replicates.

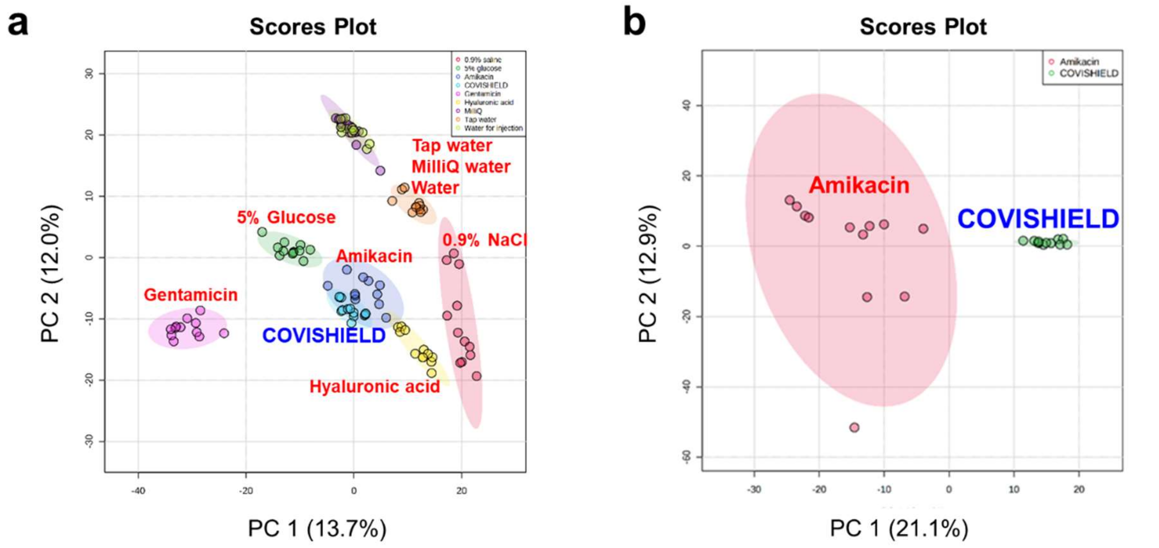

Supplementary Figure 5. PCA 2D scores plot of the Biotyper MALDI-ToF MS data for the 0-900 *m/z* mass range. **a** COVISHIELD™ vaccine as compared to 8 common falsified vaccine constituents and **b** COVISHIELD™ compared to Amikacin only.

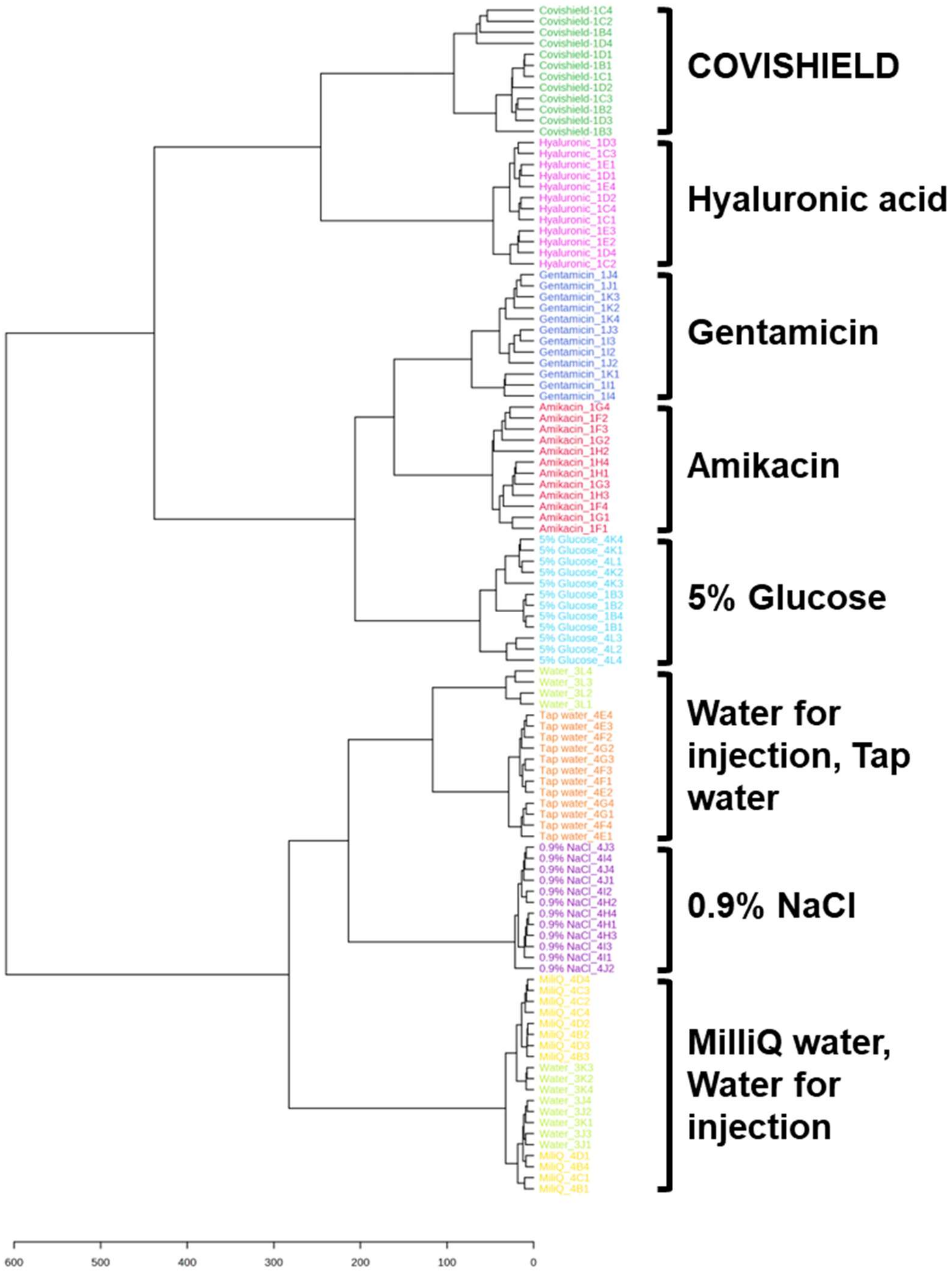

Supplementary Figure 6. Dendrogram of the Biotyper MALDI-ToF MS spectra at 0 – 900 *m/z* for the COVISHIELD™ vaccine as compared to eight surrogates of falsified vaccines.

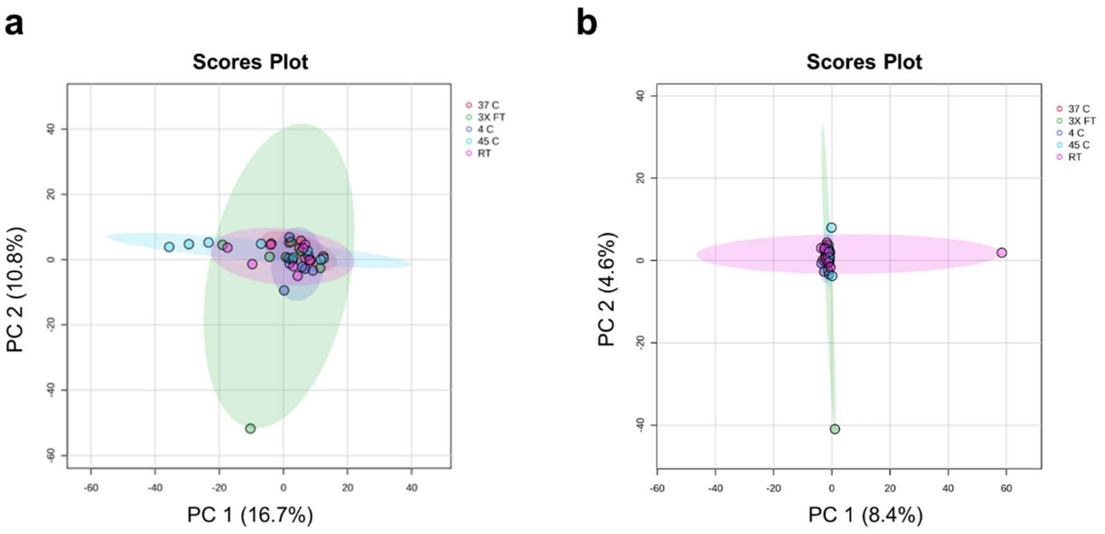

Supplementary Figure 7. Vitek-MS PCA scores plot analysis results at **a** 700-2,500 *m/z* and **b** 2,000-20,000 *m/z* comparing COVISHIELD™ vaccine vials exposed to freeze-thaw cycles and different temperature conditions (N = 8).

Supplementary Table 1. External validation and confusion matrix analysis results for PLS-DA data from Fig. 6c comparing COVISHIELD with surrogates of falsified vaccines using a 90/10 split of training/test parameters.

Full dataset

	0.9% NaCl	5% Glucose	Amikacin	COVISHIELD	Gentamicin	Hyaluronic Acid	MilliQ Water	Tap Water	Water p.i
0.9% NaCl	12	0	0	0	0	0	0	0	0
5% Glucose	0	12	0	0	0	0	0	0	0
Amikacin	0	0	12	0	0	0	0	0	0
COVISHIELD	0	0	0	12	0	0	0	0	0
Gentamicin	0	0	0	0	12	0	0	0	0
Hyaluronic Acid	0	0	0	0	0	12	0	0	0
MilliQ Water	0	0	0	0	0	0	12	0	0
Tap Water	0	0	0	0	0	0	0	12	0
Water p.i	0	0	0	0	0	0	8	0	4

Test set

	0.9% NaCl	5% Glucose	Amikacin	COVISHIELD	Gentamicin	Hyaluronic Acid	MilliQ Water	Tap Water	Water p.i
0.9% NaCl	2	0	0	0	0	0	0	0	0
5% Glucose	0	2	0	0	0	0	0	0	0
Amikacin	0	0	2	0	0	0	0	0	0
COVISHIELD	0	0	0	2	0	0	0	0	0
Gentamicin	0	0	0	0	2	0	0	0	0
Hyaluronic Acid	0	0	0	0	0	2	0	0	0
MilliQ Water	0	0	0	0	0	0	2	0	0
Tap Water	0	0	0	0	0	0	0	2	0
Water p.i	0	0	0	0	0	0	0	0	2

Training set

	0.9% NaCl	5% Glucose	Amikacin	COVISHIELD	Gentamicin	Hyaluronic Acid	MilliQ Water	Tap Water	Water p.i
0.9% NaCl	10	0	0	0	0	0	0	0	0
5% Glucose	0	10	0	0	0	0	0	0	0
Amikacin	0	0	10	0	0	0	0	0	0
COVISHIELD	0	0	0	10	0	0	0	0	0
Gentamicin	0	0	0	0	10	0	0	0	0
Hyaluronic Acid	0	0	0	0	0	10	0	0	0
MilliQ Water	0	0	0	0	0	0	10	0	0
Tap Water	0	0	0	0	0	0	0	10	0
Water p.i	0	0	0	0	0	0	0	0	10

Supplementary Table 2. External validation and confusion matrix analysis results for PLS-DA data from Fig. 8b comparing different degradation conditions of COVISHIELD™ vaccine using a 90/10 split of training/test parameters.

Full dataset

	37°C	3X FT	4°C	45°C	Boiled	RT
37°C	12	0	0	0	0	0
3X FT	0	12	0	0	0	0
4°C	0	1	11	0	0	0
45°C	0	0	0	12	0	0
Boiled	0	0	0	0	12	0
RT	0	0	0	0	0	12

Test set

	37°C	3X FT	4°C	45°C	Boiled	RT
37°C	2	0	0	0	0	0
3X FT	0	1	0	0	1	0
4°C	0	1	1	0	0	0
45°C	0	0	0	1	1	0
Boiled	0	0	0	1	1	0
RT	1	0	0	0	0	1

Training set

	37°C	3X FT	4°C	45°C	Boiled	RT
37°C	10	0	0	0	0	0
3X FT	0	10	0	0	0	0
4°C	0	0	10	0	0	0
45°C	0	0	0	10	0	0
Boiled	0	0	0	0	10	0
RT	0	0	0	0	0	10

Supplementary Table 3. External validation and confusion matrix analysis results for PLS-DA data from Fig. 9c comparing genuine COVISHIELD vaccine vial label, office stationery label, and matrix using a 90/10 split of training/test parameters.

Full dataset

	COVISHIELD	Matrix	Office label
COVISHIELD	48	0	0
Matrix	0	8	0
Office label	0	0	8

Test set

	COVISHIELD	Matrix	Office label
COVISHIELD	5	0	0
Matrix	0	1	0
Office label	0	0	1

Training set

	COVISHIELD	Matrix	Office label
COVISHIELD	43	0	0
Matrix	0	7	0
Office label	0	0	7

Supplementary Table 4. External validation and confusion matrix analysis results for PLS-DA data from Fig. 10b comparing vaccine vial labels from two different sites of vaccine manufacture using a 90/10 split of training/test parameters.

Full dataset

	Hadapsar	Manjari
Hadapsar	40	0
Manjari	0	8

Test set

	Hadapsar	Manjari
Hadapsar	4	0
Manjari	0	1

Training set

	Hadapsar	Manjari
Hadapsar	36	0
Manjari	0	7
